# A necklace of calcifications: a rare clinical image

**DOI:** 10.11604/pamj.2025.50.49.46640

**Published:** 2025-02-12

**Authors:** Gaurang Aurangabadkar, Sumer Choudhary

**Affiliations:** 1 Department of Respiratory Medicine, Datta Meghe Medical College, Nagpur, Datta Meghe Institute of Higher Education and Research (DMIHER), (Deemed University), Sawangi (Meghe), Wardha, Maharashtra, India

**Keywords:** Tuberculosis, lymphadenopathy, calcifications

## Image in medicine

A 72-year-old female presented to the respiratory physician with chief complaints of dyspnea at rest and chest pain present for the last 8 months. The patient gave a history of lymph node tuberculosis 6 years back, for which she took irregular treatment. A chest X-ray postero-anterior (PA) view was done which revealed the presence of extensive mediastinal and pulmonary calcified lymphadenopathy. The patient was started on symptomatic treatment along with oxygen support for low oxygen saturation levels and was discharged after 10 days. Lymphadenopathy is one of the cardinal features of primary tuberculosis. The initial focus of infection, known as Ghon´s focus can usually be seen in the lower lobes of the lungs, after which the mycobacterium spreads to the regional lymph nodes. This process may get enhanced as the disease progresses and during the healing phase, calcification of the mediastinal lymph nodes is usually seen. Extensive mediastinal calcification can be found in rare cases and is considered to be a chronic complication of healed tuberculosis infection.

**Figure 1:**
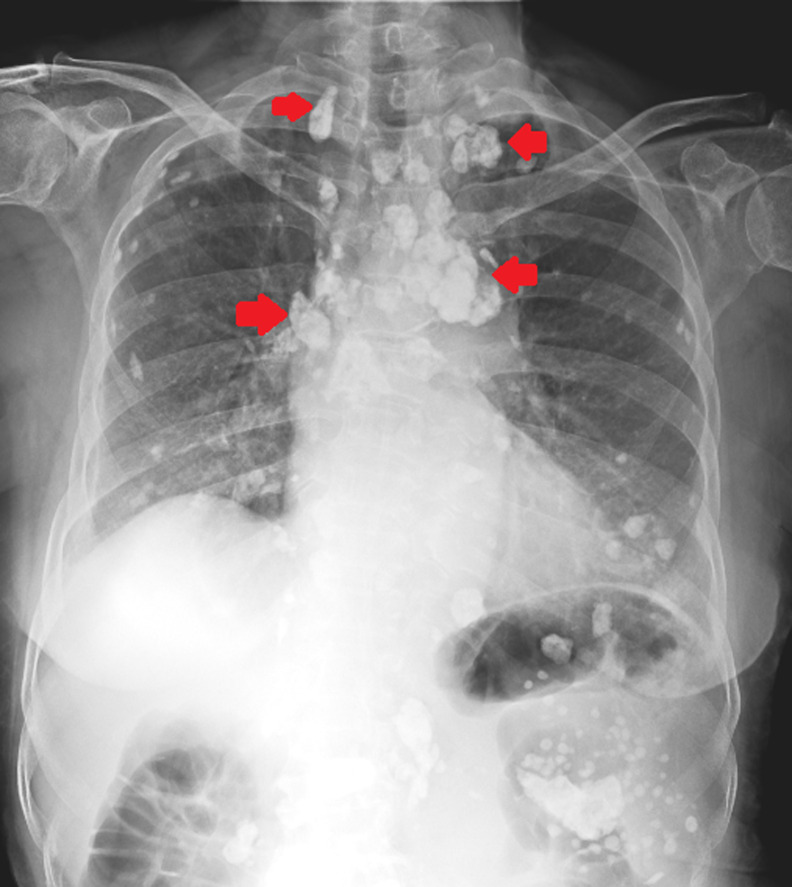
chest X-ray postero-anterior (PA) view showing extensive mediastinal calcifications (red arrows) with diffuse lung calcifications secondary to lymph node tuberculosis

